# Supporting family carers in Ireland: the role of the general practitioner

**DOI:** 10.1007/s11845-022-03031-9

**Published:** 2022-06-15

**Authors:** Mary Cronin, Sinead McGilloway

**Affiliations:** Centre for Mental Health and Community Research, Department of Psychology and Social Sciences Institute, Maynooth University, Maynooth, Ireland

**Keywords:** Family carer, General practice, Health and wellbeing, Ireland, National Carer Strategy

## Abstract

**Background:**

Ireland has over half a million family carers who provide care to a family member or loved one. Internationally, it is recognised that general practitioners (GPs) have a critical role to play in the identification and support of family carers, but, to date, no guidelines exist in Ireland to support GPs in this role.

**Aims:**

The aim of this study was to examine how carers are currently supported (or not) by healthcare professionals in Ireland, with a particular focus on the role of the GP.

**Methods:**

A mixed method design was used, involving a national online survey (*N* = 132) of family carers in Ireland and one-to-one interviews with 10 stakeholders (4 GPs; 6 carers). The quantitative data were analysed using a series of descriptive and inferential statistics; the interview data were analysed using framework analysis.

**Results:**

Sixty-one per cent of the carer sample reported experiencing psychological distress, more than two-thirds of whom (69%) reported ‘rarely’ or ‘never’ being asked about their own health and wellbeing. Sixty-one per cent also felt misunderstood in terms of the challenges they face in their caring role. Three key themes were identified from the interview data including (1) GP role ambiguity; (2) navigating informal processes and (3) changing needs along the care trajectory.

**Conclusions:**

The findings suggest important gaps in terms of the role of GPs vis-à-vis their support of family carers. GPs themselves indicated that they need both greater clarity regarding their role with family carers and more training and resources in this regard. A requirement for more streamlined communication and information provision was also highlighted by both GPs and carers. Carers reported a need for more information on the role of GPs in supporting carers as well as more support in addressing, in particular, the psychological complexities of carer identity and help seeking.

## Introduction

Care needs are increasing in our society due to improved longevity, advances in medical care and a shift away from institutional care [1]. Eighty per cent of long-term care (LTC) in Europe is provided by family carers [2] while recent Central Statistics Office [3] figures from Ireland indicate that 1 in 8 people over the age of 15 is providing care to a family member [4]. Although caring [5] has been reported to have some benefits for carers (e.g. a sense of purpose and achievement), greater caring responsibilities have been linked with progressively poorer health outcomes for carers [6]. Indeed, those providing care often experience negative consequences to their own health and wellbeing. For example, a recent survey of family carers in Ireland (*N* = 1250) found that 45% reported a long-term illness, health problem most of whom (80%) felt that their caring responsibilities had contributed to their illness/disability [4]. These figures are also reflected in other work undertaken internationally [7, 8], thereby indicating that large numbers of family carers require more health and wellbeing support.

It has been suggested for decades that community health professionals such as general practitioners (GPs), public health nurses/health visitors and primary care centres are well placed to support carer identification, health and wellbeing [9, 10]. Furthermore, family carers have more contact with their GP than any other health professional [11]. For example, a recent international scoping review of physicians’ perspectives of their role in supporting family caregivers indicates that primary care is the appropriate context for identifying and supporting carers [10]. However, barriers still exist at practice, health systems and policy level. For example, existing evidence points toward a number of factors that impact on adequate carer identification and support, including lack of time and reimbursement; failure to self-identify as a carer; focusing on the care recipient to the exclusion of the carer (by both the carer and the health service provider); disjointed health and community systems; inadequate services; and a lack of policy and ethical guidance [10]. The importance of primary care in the identification and support of carers was also highlighted in another scoping study involving a diverse sample of professional stakeholders in the UK [12]. The findings indicate that the failure of carers to self-identify, or to recognise themselves as carers and the ambiguity within primary care services to proactively identify carers, were key obstacles to the provision of appropriate, effective and timely carer support.

An analysis of responses from carers in the 2011–2012 English general practice survey (*N* = 195,364) showed that they reported lower health-related quality of life (HRQoL) when compared with non-carers, and this was especially marked amongst those providing longer periods of care [6]. Carers also reported poorer patient experience than their non-carer counterparts with regard to access, making appointments, seeing their preferred doctor, receptionist communication, doctor and nurse communication, and overall experience. HRQoL in carers and carer burden have also recently been explored in the context of health literacy (HL), with high levels of health literacy found to be significantly associated with lower carer burden [13]. Other research in this regard has highlighted the importance of improving communication between carers and healthcare professionals and recommended that, for example, a short assessment be used to guide consultations [14] and/or that communication skills training for carers [15] and GPs [16, 17] may be beneficial.

GPs in Ireland, as elsewhere, play a key role in healthcare delivery, and as is the case internationally, demand is increasing and expected to increase further, leading to workload concerns in general practice [18]. Consequently, in order to support family carers, GPs need effective streamlined resources to support them in this task. Some guidance and training are available to support GPs in their role vis-à-vis family carers, in a number of western countries (e.g. UK, Australia and Canada) [19, 20]. However, no such guidelines are as yet available in Ireland to support GPs or other health professionals despite the fact that the National Carers Strategy [21] — which represents an important first step toward recognising the contribution of family carers and supporting them in their role — calls for more effective approaches to identifying and supporting family carers in healthcare settings.

The current study was conducted as part of a larger project (called ‘CHERISH’) designed to raise awareness amongst GPs of the physical and emotional health issues experienced by carers and to identify how best to support GPs and promote more ‘proactive approaches to the identification of carers’ [21] (p. 20). The objectives of the study reported here, were to: (1) examine how carers are experiencing access to supports in relation to their own health and wellbeing, with a particular focus on psychological barriers to help seeking; and (2) assess GP perceptions of carers and their caring role in order to better understand the processes by which they identify carers and the extent to which they support (or not) carer health and wellbeing.

## Methods and procedure

This study used a two-stage explanatory sequential mixed method design [22]. Stage one involved the design and administration of an online national survey of carers, whilst stage two entailed a small number of in-depth one-to-one interviews with both carers and GPs.

### Participants and settings

A convenience sample of carers was recruited online through social media platforms (Twitter and Facebook), where specific pages were created for the research and a link to the online survey was provided. Participants were required to be over 18 and to be providing care to a family member or loved one. On completion of the survey, respondents were invited to express an interest in taking part in stage two of the study. GPs were recruited for stage two via a forum post which was placed on a website resource for GPs in Ireland called GPbuddy.ie (https://www.gpbuddy.ie).

#### Stage one: online survey

The Family Carer Questionnaire (FCQ) was developed specifically for the purposes of this study and included a number of psychometrically robust measures (see below), as well as a brief background questionnaire comprising a number of sociodemographic and background items (e.g. age, gender, reason for providing care, duration of caring role, caring hours) combined with several Likert-style questions on identifying as a carer, experiences with healthcare professionals (HCPs) and engagement with activities that may help/support carers in their caring role. The last of these was adapted from the caregiving-related activities scale which was used in the large American Association of Retired Persons (AARP) caregiver identity study (*N* = 4037) in the USA [23]. Four open-ended questions were also added to inquire about the nature, availability and impact of health and wellbeing supports provided (or not) by GPs and other HCPs as well as respondents’ feelings about being referred to as a ‘carer’ and their own help seeking behaviour.

The 12-item version of the General Health Questionnaire (GHQ-12) was included to assess overall psychological distress or minor psychiatric morbidity [24]. Items were scored using the Likert method 0–1-2–3 as recommended by Goldberg (1997), with total scores ranging from 0 to 36. The scoring thresholds are as follows: a score of 1–10 indicates ‘low psychological distress’; 11–12 is ‘typical’; 13–15 is ‘more than typical’; 16–20 shows ‘evidence of psychological distress’ and scores over 20 indicate ‘severe distress’ (Goldberg et al. 1997).

The 40-item Adult Carer Quality of Life Questionnaire (AC-QoL) was used to measure quality of life (QoL) in eight separate domains including ‘caring choice’, ‘money matters’, ‘support for caring’, ‘caring stress’, ‘personal growth’, ‘sense of value’, ‘ability to care’ and ‘carer satisfaction’. Domain scores ranging from 0 to 5 indicate a low reported quality of life, 6–10 indicate a mid-range reported quality of life and 11 + indicate a high reported quality of life [25]. Total scores range from 0 to 120 with higher scores indicating better QoL and categorised as follows: low (0–40), mid-range (41–80) or high (81 +).

The FCQ was piloted with five carers from different care situations (i.e. parent caring for a child, son/daughter caring for parent, spouse carer), after which some minor adjustments to layout and wording were made. The data were analysed using SPSS; all open-ended questions were analysed ‘semi-qualitatively’ to identify key categories/themes.

#### Stage two: in-depth interviews

A series of semi-structured interviews was conducted with six carers and four GPs as part of stage two. Two separate interview schedules were devised on the basis of a review of the literature, and piloted with a GP and carer respectively in order to ascertain the appropriateness of content, wording and timing. Due to COVID-19 restrictions at the time of the study (2020), it was not possible to conduct face-to-face interviews. Furthermore, the recruitment of GPs for research can be extremely challenging due to the fact that they are time poor [26], as are many carers, and both groups had significant additional and new demands placed upon them during the restrictions and lockdowns of the COVID-19 pandemic [27, 28]. Telephone interviews were therefore considered to be a convenient and time-efficient method of data collection that did not require any additional software or technological know-how. These have also been successfully used in the past as a convenient and efficient means of interviewing GPs [29, 30].

All interviews were audio recorded with consent, anonymised and transcribed verbatim in preparation for analysis, which was conducted using NVivo. Framework analysis, which is widely used in medical and healthcare research, was used to analyse the data [31]. This involved a five-stage process of: (1) initial familiarisation with the data; (2) identifying a thematic framework; (3) indexing; (4) charting; and (5) mapping and interpretation [32].

## Results: stage one

### Participant profile

A total of 132 carers from 23 counties in Ireland and from an almost equal mix of urban and rural locations, participated in the survey. Respondents were predominantly female (89%) with a mean age of 50 (SD = 10.57), were caring for a family member/loved one for an average of 11 years (SD = 11.62) and spent 17 hours in any typical 24-hour period in a caring role (SD = 7.40) for an average of 6.6 days per week (SD = 1.05). The mean age of the care recipient was 51 (SD = 31.64) and reasons for providing care were multiple and complex with 11% of the sample providing care for more than one person. Many carers reported that the care recipients had multiple co-morbidities, such as physical and intellectual disabilities, autism and intellectual disability and dementia often present with other complications of senior care.

### Health and wellbeing

**Fig. 1:**
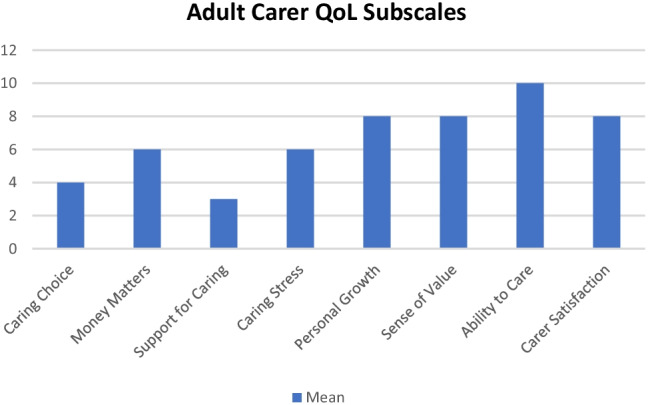
Adult Carer Quality of Life subscale scores*. *(0–5 indicates a low reported quality of life and may suggest problems or difficulties; 6–10 indicates a mid-range reported quality of life; 11 + indicates a high reported quality of life)

Sixty-nine per cent of respondents were ‘rarely’ or ‘never’ asked how they were; only 3% reported that they were ‘often’ asked this question. The carer sample obtained a mean score of 23 (SD = 6) on the GHQ-12 (Cronbach alpha 0.86), indicating typically severe levels of psychological distress (Goldberg et al. 1997). No significant differences were found by gender (*p* = 0.72) or community setting, i.e. rural or urban (*p* = 0.39), although a correlation analysis revealed a small negative correlation between total GHQ-12 scores and age (*r* =  − 0.207, *n* = 112, *p* < 0.05), with younger carers reporting higher levels of psychological distress. The total mean score on the AC-QoL (Cronbach alpha 0.78) measure was 55 (SD = 16), indicating QoL levels in the lower to mid-range (mid-range, 41–80). No significant differences were found by gender (*p* = 0.97), age (*p* = 0.74) or community setting (*p* = 0.18). An analysis of the eight subscales showed that the lowest scores were reported for ‘Support for Caring’ (Mn = 3, SD = 2) and ‘Caring Choice’ (Mn = 4, SD = 3), suggesting that carers perceived a low level of emotional, practical and professional support and a low level of control over their own life. Conversely, respondents reported the highest mean score on their ‘Ability to Care’ (Mn = 10, SD = 3) (Fig. 1), indicating high levels of competency to care.

**Fig. 2:**
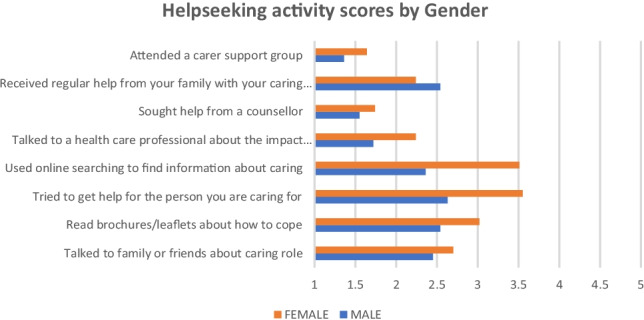
Help Seeking Activities by gender*. *Likert scoring: 1 = ‘Never” and 5 = ‘Often’

More than three-quarters (79%) of carers reported that they had little or no support to help them in looking after their own health and wellbeing, and 85% (93/110) indicated that this had a negative impact on their health and wellbeing. This was also reflected in the mean score of 20 (SD = 6) on the ‘engagement with help seeking activities’ items devised for this study (Cronbach alpha 0.65), which indicated a generally low level of engagement, but with females reporting significantly more engagement than males *(p* = 0.035, *r* = 0.2) (Fig. 2). These findings were amplified in the responses provided by most of the sample to an open-ended question around help seeking, which showed that only 11% (9/82) were comfortable to ask for help, whilst an identical proportion felt that help would not be forthcoming, so they decided not to ask. The remainder of respondents either had difficulty in asking for help (41%, 34/82) or stated that this was something they would never do (28%, 23/82).

## Understanding from healthcare professionals

Respondents (61%) felt that HCPs, including GPs, rarely if ever understand the challenges they face in their caring role, while approximately half (51%) felt the same way in relation to HCP concern for carers’ own health and wellbeing. More than three-quarters (77%) felt that HCPs are ‘rarely’ or ‘never’ interested in hearing about their experiences of caring. Further analysis showed a small statistically significant positive correlation between the number of years spent caring and perceptions of understanding from HCPs (*r* = 0.20, *p* < 0.05), whereby those who had been carers for longer, felt less understanding and concern from HCPs. Responses to an open-ended question which explored this further, showed that 40% of those who responded (38/95), reported that they would value practical supports such as referral to support agencies, advocacy regarding respite/home support, and information. A similar proportion (38%, 36/95) indicated they would like to be asked how they are, and to be listened to, whilst 9% (9/95) were unsure what HCPs could do for them, with 4% suggesting counselling. Only 3% were happy with the supports they were receiving.

### Identifying as a carer

Just over one-quarter of the sample (27%) indicated that they would describe themselves as a ‘carer’ whilst over half (53%) ‘rarely’ or ‘never’ referred to themselves as a carer when completing official documents. Further responses to an open-ended question in this regard, showed that approximately one-third (34%, 37/108) disliked being referred to by HCPs as a carer due to, for example, a perception that this diminished their familial role and identity. However, the largest proportion (59%) either did not mind the label (31%, 34/108), or felt it was a validation of the care they provided (28%, 30/108); 6% stated that they had never been asked about their caring role.

### Stage two

Interviews with carers lasted approximately 1 hour and with GPs, approximately 30 min. Carers had a range of caring roles (Table 1), and had been caring for their relative for periods ranging from 3 to 18 years.

**Table 1:** Carer participant characteristics

***Relationship to care recipient***	***Reason for care***	***Years caring***
Mother	Autism	18 years
Husband	Rheumatoid arthritis	8 years
Brother	Mental health	14 years
Wife	MS	19 years
Daughter	Senior care	13 years (recently bereaved)
Daughter	Senior care	3 years

Care recipients had a mix of care needs and were aged 18 to 93. Sadly, one carer had suffered the loss of the person for whom they were caring, in the interval between completing the survey and being invited to the interview, but they were still keen to participate in the interview nonetheless. Three overarching themes were identified from the analysis, as outlined below; these are presented in order of their prominence within the data.

#### Navigating through informal processes

All four GPs reported that they were navigating an informal system of carer identification and sourcing information, a process which was compounded by issues relating to documentation and fragmentation of services. Likewise, all of the carers were navigating a system where they felt the provision of information and support was typically ad hoc, difficult to access and often covert. The findings suggest that carers are typically identified by GPs on a very informal basis and often informed by their longer experience in primary care, as well as the duration of time the care recipient has been attending their practice; longer term attendance by the care recipient led to better (informal) identification of carers and their needs, but no formal information about carers is typically conveyed to GPs. The GP interviewees reported that they may be alerted to a particular carer need on occasion (i.e. a carer struggling, a care recipient deteriorating), but this was often communicated informally by another member of the primary care team who may have noticed something and ‘flagged’ it with the GP, or the GP heard there might be an issue through another source. This type of informal ‘grapevine’ communication system was a challenge for the GPs because, professionally, it can be difficult for them to act on informal information. The GPs were then left in a situation of trying to ‘broach’ it with the carer, but doing so tentatively or waiting to ascertain if anything else would arise to support the conversation. One GP spoke of how this can be a particular problem where there may be a safeguarding concern in the caring role:

And when you are hearing it third hand and then they are presenting with the person they are caring for …. and that its quite tense….that’s when its problematic, That can be quite difficult to broach….. (GP2)

The lack of information and the fragmentation of services were a source of considerable frustration to both GPs and carers. The GPs indicated that they did not have any information about what was available in their community and this was a clear barrier to having discussions with carers about their needs:

We have no access to what’s available in the community to advise our patients… just to be able to click and go ok, we have got that or this person that we can contact. We don’t have any of it…. (GP4)

Carers reported that often, after many fruitless attempts to obtain information, they were eventually given very valid and useful information ‘unofficially’ from healthcare staff such as nurses and co-ordinators who imparted the information in an almost clandestine manner. This advice/information typically focused on how to ‘manage’ the system in order to secure the best outcome for their loved ones. While carers were very pleased to receive this information, it did leave them wondering why information had to be conveyed in this way:

And I think, who knows about these things—that’s what I don’t understand. Only that I opened my mouth to appeal it, I wouldn’t know a thing about it. (Carer 4)

I have to say in thanks to some people in the HSE [Health Service Executive, Irelands national health system] who would never tell you they told you, would never write anything down, would only tell you over the phone, but it was thanks to those people who told me. [how to access a service] (Carer 5)

Interactions with carers were not documented by GPs, other than if the carer was a patient and then only two of the four GPs interviewed, stated that they had documented the identity of the carer. For example, one GP reported inserting the carer’s name on the patient’s (care recipient’s) chart with permission, for contact and communication purposes. Another GP commented that, while he did not currently record carers’ names in the patient file (as he knew them all), he would likely do that in future in the event of having a locum filling in at the practice.

#### Role ambiguity

Ambiguity around the role of GPs in supporting carers’ health and wellbeing was a strong and recurring theme amongst interviewees. The role of the GP was seen as being of limited value for the carer unless they had a particular ‘medical problem’ to discuss, or required paperwork or medication management for their relative/loved one. There was a sense that the GP could not help with many of the challenges of caring, such as accessing appropriate services. There was also an acute awareness and appreciation from the carer’s perspective, of the busy primary care environment, and they felt that the GP would not have the time for them to discuss their caring-related concerns. However, the carers’ assessment of their own needs was mitigated somewhat by making comparisons to the difficult circumstances of other carers whom they knew and the complex medical needs of the care recipients. Therefore, they often hesitated in raising their own needs with their GP as they were unsure as to whether or not it was appropriate, or if they would be justified in so doing:

…but you see, I don’t know is the GP the way to go…but you know you don’t know who to go to in a way? (Carer 4)

There’s no point in seeing the GP they haven’t got anything, they have nothing for you……it was all the paperwork we went in with this form or that form for whatever it was. It was very practical you know……. like it’s not a medical problem. (Carer 5)

Notably though, those who had longstanding relationships with their GPs were more comfortable with discussing their own needs, and spoke highly of the support they received.

The caring role was typically portrayed (by both GPs and carers) as being adversely affected by decisions made within the wider health service system, often without consultation with the carer. Carers felt that while frontline staff and, in particular, home support carers, disability services staff, and palliative care nurses were often very understanding of their role and went above and beyond to support them, there was much less understanding at higher levels of the health service (e.g. policy makers, home support co-ordinators). In some instances, this was an important barrier to carers communicating their needs. For example, one carer spoke of how a GP suggested that she learn how to manage and replace a catheter herself rather than calling a healthcare professional as she ‘would be well able for it’; likewise, another spoke of how a PHN had suggested that they were ‘well able’ and left them to tend to dressing wounds themselves. References to carers as being ‘able bodied’ or ‘capable’ were used as a means to encourage them to take on extra caring responsibilities, but without any assessment of their current circumstances or capacity to care.

GPs also felt some ambiguity about their role with carers. None of the GP interviewees had any awareness of the National Carers Strategy or its objectives regarding the support of carers in health services including, general practice. Although they were sympathetic to the needs of carers, they were very much working with their own intuition regarding if, and how, they should support carers, with some making efforts to source supports while others did not feel this was their responsibility.

So obviously I’m involved with the PHN and I’m providing medical and mental health services to the carer, so after that not a lot else really, if there are support groups they will find those themselves I’m not that familiar with what’s involved there. (GP 2)

I think that something is definitely lacking in primary care … I let them call the places, and I say ‘if you need anything from me’ to back this up let me know…it’s a time limit thing really, but also from a general practice point of view, we are not aware of what’s out there. (GP 4)

All of the GPs agreed that additional resources and/or training would be necessary in order for them to more effectively support carers. For example, they provided a number of helpful suggestions in this regard, including an up-to-date database of specific carer resources and easily accessible and short training (e.g. by means of short videos and/or through local GP group meetings).

#### Changing needs along the care trajectory

The caring role was identified as ever-changing and evolving over time, with numerous different challenges. Although it was not addressed explicitly in the interview schedule, all of the carers spoke about the time their loved one was first diagnosed. This clearly had a considerable emotional impact on them and was remembered as a hugely stressful time where they felt they were given little information or support; furthermore, all stated that it took them years to negotiate their way through the process of obtaining appropriate support for their loved one. Conversely, challenges for carers at the time of diagnosis were not raised by any of the GP interviewees.

The GPs referred to family support as a possible source of help for carers, although the experience of the carers was mixed in this regard. Those who had strong family support greatly benefitted from it, but carers who had very difficult caring responsibilities (e.g. MS, severe autism) felt that their families did not fully understand their situation and had distanced themselves from them over time. As caring progressed, accessing informal help from family or friends often became more challenging, even if family had previously been providing help. Furthermore, when the caring circumstances progressed to a requirement for specialised care, carers reported that this could only be delivered by someone who was appropriately trained.

His disability, his illness has progressed over time. It’s a long term chronic illness—it’s not just going to go away tomorrow…this is for the long haul…And that’s the hardest part….I just find friends diminish very fast with disability, with illness (Carer 6)

They [adult children] are working and everything you know, like, I mean they have things too……you don’t want them to be torn between, you know looking after somebody and you know, trying to do a job themselves and look after their own families. (Carer 4)

Key transitions on the caring ‘journey’, such as the care recipient moving to residential care or approaching end of life, were periods of additional stress for carers, during which they reported a need for higher levels of support. Several of the carers spoke about how the progression of the caring role over time, meant that they had to stop working or work shorter hours. Changes to services for the care recipient throughout the care trajectory also had a significant impact on carers, with several alluding to how cuts to, or withdrawal of, services, had negatively affected their own ability to work or to have any kind of social life for themselves.

## Discussion

This study was conducted to investigate how carers in Ireland are supported in relation to their own health and wellbeing needs, and to explore GP perceptions of carers and the processes by which they identify carers (or not) and support their health and wellbeing. The results showed that the vast majority of family carers were rarely if ever asked how they were, despite the fact that most were clearly in need of formal mental health intervention according to their GHQ scores. The in-depth interviews helped to shed light on some of the reasons why this might be, particularly with regard to their attendance at primary care. As shown in research conducted elsewhere [21], the GPs were ambiguous about their role vis-à-vis carers and this was particularly true if the carer was not a registered patient of the GP practice and if the GP had only limited information about them. Furthermore, when the topic was raised in conversation, the GP interviewees felt that they had little or no information about the services available for carers in their area, while patchy service provision was also a source of considerable frustration for them. This is in line with the findings of a recent scoping view by Parmar et al., indicating that a disjointed health and community system can impact carer identification and support [10]. The carers in the current study, likewise, were unsure about how GPs could best support them, and this perception was a key barrier to their help seeking. Importantly, some also were reluctant to seek help, or to communicate their concerns, as they felt this could further burden GPs or other HCPs in their role. Indeed, a recent survey of members of the Irish College of General Practitioners (ICGP), found that GPs in Ireland carry out an average of 29 consultations a day and report higher levels of exhaustion than their counterparts in Europe and the UK [33]. Our findings suggest that these demands may influence the extent to which (and how) some carers (and possibly patients) approach concerns about their health in primary care settings.

The identification of carers in general practice is a logical first step in providing support, but this remains a complex issue, with a number of barriers from both a carer and GP perspective. Recommendations from research and guidelines internationally have aimed to address how GPs might identify carers through, for example, practice initiatives [19, 34, 35] and/or the provision of carer-focused training [36]. The introduction of carer-focused initiatives in GP practices (e.g. appointing a member of staff to act as carer champion and with primary responsibility for identifying carers) has been recommended in the UK by a number of organisations, including the Royal College of General Practitioners (RCGP), Carers Trust Wales and the National Institute for Health and Care Excellence (NICE) [34, 37]. Our findings suggest that GPs in Ireland (and possibly also elsewhere) would benefit from brief training and additional resources to support them in their role with carers. For example, an interesting pilot study of GP training in England, found a positive impact of training across many areas, including better knowledge of carers, increased awareness of need and greater GP confidence in supporting carers [36]. The formal assessment of carer needs in general practice has also been shown to be valuable in identifying overall levels of need and guiding the consultation process [14, 34, 38].

Importantly, our findings highlight the many psychological complexities for carers regarding their own needs, including carer identity, understandings and perceptions around self-care and communication with GPs (and other HCPs), all of which also need to be addressed. The nature and extent of these barriers, evident from our findings, suggest that encouraging self-identification amongst carers may not be sufficient, and that they may also benefit from psychological interventions that would help them to explore identity, self-care and communication skills specific to healthcare settings. Indeed, communication-enhancing interventions have been shown to be effective in healthcare settings, both when used by HCPs and patients alike [39], and recent work on carer burden has also highlighted health literacy to be a factor [13]. A communication and empowerment intervention for carers, could also be helpful in terms of enabling them to explore carer identity and support them in having difficult conversations with their doctor about the impact of caring, their own health needs and concerns about the person for whom they are caring. However, previous research suggests that barriers exist, not only at individual carer level, but also within practices [40], and therefore, any carer-focused initiatives at practice level may only have a limited impact in the absence of individual supports for carers themselves.

The number of carers in Ireland is estimated to be in excess of half a million [4]. However, our findings indicate that many carers do not refer to themselves as such when completing official documents, thereby suggesting that these figures are likely to be extremely conservative. A recent working paper on family carer enumeration, [41] suggests that several issues exist in our current recording of carers in Ireland, resulting in likely under-reporting. These include challenges such as irregularities in the carer data, including the low numbers reported through the Census of Population. For example, in Census 2002 and 2016, the reported occurrence was 4.8% and 4.1% respectively. These figures are very low compared to, for example, Northern Ireland where 12% of the population in 2011 identified as carers. The Irish Health Survey, a large nationwide survey administered by the Central Statistics Office, also gathers data on carer numbers, and reported a similar figure in 2015. Such variation may be due to how the questions in surveys are worded and/or interpreted but also, as shown here, the extent to which carers identify as such.

As outlined in our previous work [42], identifying as a carer has clear implications for seeking help or support. Typically, however, it is not until later in the caring trajectory that those who provide care, identify with the term carer and seek support and often this will not occur until a point of crisis [43, 44]. The results reported here, suggest that early intervention is important, as previously pointed out by Carduff et al. in the UK [40], while the point of diagnosis may also be an ideal time to identify who will be providing the care [45]. In Australia, the Northern Sydney Health District guidelines, ‘Think Patient, Think Carer’, suggest that the carer should be identified at first appointment or at the point of diagnosis [20]. Caring challenges for the carers in our study, clearly emerged from the point of diagnosis of the persons for whom they were providing care, but at this very crucial stage, they did not know where to turn for support. While carers may not be ready to accept help at an early stage, it is important, nonetheless, that they are alerted to existing supports, including those available through their GP, so that they know from whom to seek assistance when/if the need arises.

The GPs in the current study reported a lack of information regarding resources for family carers in the community. This is interesting because they were recruited through ‘GP buddy’, an online resource for GPs and other healthcare professionals in Ireland, providing information about other medical professionals and services. Perhaps consideration could be given to the inclusion of community-based resources or social prescribing options on platforms used routinely by GPs when seeking local or national information. The results reported here, also highlight the lack of awareness amongst the GP participants, of Ireland’s National Carers Strategy (NCS) [21], thereby suggesting that perhaps greater efforts are needed to communicate policy objectives to frontline staff and key stakeholders. Indeed, this would also help with policy implementation more generally. In the UK, the RCGP include a reference in their carer guidance document (‘Supporting Carers: An action guide for general practitioners and their teams’ (p. 15)), to the UK Carer Strategy, and the key elements therein that GPs might address [46]. A recent scoping review by Parmer et al. further highlights reimbursement as a barrier to the identification and support of carers in general practice [10]. The UK quality and outcomes framework (QOF) that is intended to compensate general practices for providing good quality care provides financial rewards for GPs for stipulated carer care [47]. This was not something that was raised by GPs in this study and, to date, has not been addressed at policy level in Ireland.

Worryingly, some of the carers in this study also reported that they were expected to complete ‘medicalised’ tasks. Concerns regarding increased medicalised tasks by family carers, have recently been raised elsewhere. For example, the American Association of Retired Persons (AARP) highlighted the increasing amount of complex care being provided in the home, in a recent ‘Home Alone Revisited’ study (*N* = 2089) [48]. Over half of the carers in this study were engaging in medical/nursing tasks that had previously been carried out by HCPs, with 7 out of 10 of these carers dealing with pain management. Furthermore, those engaged in these tasks, reported more time spent caring and a heavier emotional responsibility. Dow et al. refer to the shift of complex medical care to the community as an ‘invisible contract’, whereby carers are expected to assume a high level of responsibility for care tasks in the home that were previously the work of paid staff [49]. Arguably, the setting of clear parameters with regard to family caring, should be carefully considered and agreed amongst all parties so that carers are not expected to provide care that should be the responsibility of medically trained professionals.

Our findings highlight further an urgent requirement for the assessment of carer needs, particularly around their capacity to care, and their need to avoid the, often overwhelming, burden of shouldering additional responsibilities. Notably, White et al. recommend that a ‘Carer Readiness Tool (CRT)’ should be used before hospital discharge in order to assess carer readiness to undertake the caring role and to help HCPs assess the limits of what carers can be expected to do, as well as engaging them in discharge planning [50]. Our findings suggest that a similar tool could be usefully employed in settings in Ireland, and with routine in-built monitoring and review. This is particularly important in view of a number of societal changes to suggest that the frequency and complexity of care offered in the community are likely to increase in the not-too-distant future, owing to a generally ageing population (who are living longer), the shift away from institutional forms of care and an increased emphasis on home care [1]. It is imperative, therefore, that family carers are not unduly burdened with a level of care that would historically be carried out by HCPs.

## Strengths and limitations

This study addressed, for the first time in Ireland, the nature and extent of support provided to family carers in healthcare settings, with a particular emphasis on general practice. The study employed a mixed method design and explored the barriers and facilitators to HCP identification and support of carers, including GPs. This is important in light of some of the objectives outlined in the National Carers Strategy in Ireland which, to date, have not been fully implemented. The one-to-one interviews, whilst based on only a small sample of carers and GPs, yielded some interesting and important insights into the complexities surrounding the challenges involved in meeting these policy goals. The online administration of the survey also circumvented the need to recruit the sample using more traditional social welfare department or support services, and this may have encouraged greater participation amongst those who do not usually identify as carers. This is an important lesson in terms of carer recruitment which is typically based on convenience sampling of those who are in receipt of carers allowance and/or who have registered with a carer organisation [4, 51], thereby only reaching those who explicitly identify as a carer.

The findings from this study were important in informing the research questions underpinning an international scoping review of the literature that was subsequently undertaken as part of the larger CHERISH project. This was carried out to determine the nature and extent of any guidance available internationally to GPs to assist them in consultations with carers as part of their day-to-day role. The results reported here have also been used to inform the development and pilot testing of a brief training workshop for GP registrars (with an accompanying suite of supportive materials) to help them better identify and support family carers.

At the same time, the study was limited in a number of ways. The survey sample was not large, although it comprised a very diverse group of carers drawn from 23 of the 26 counties in Ireland and across a wide range of age and care recipient needs. It is possible that only those carers who were most adversely affected by their caring role decided to participate in the survey and interviews, and indeed, the lack of information on non-respondents is a well-known drawback of the survey method [52]. Male carers were underrepresented in the sample despite vigorous efforts to recruit more men through engagement with the Men’s Health Forum Ireland (who shared the survey link with members) and with Kilbeggan Mens’s Sheds in the Midlands. Unfortunately, the low participation by males is common in carer (and other) research [27, 53]. Furthermore, the GPs who participated, did so because they had a particular interest in carers due to past professional or personal experiences and they may not, therefore, be representative of GPs who have not had such experiences. Additionally, they were all recruited through the same GP online forum, and so it is difficult to know the extent to which they were representative of all platform users. Lastly, the onset of the COVID-19 pandemic in March 2020, meant that fewer interviews were conducted at that time than anticipated and before saturation could be reached. Attempts to contact further participants were unsuccessful and it was felt that, due to the unprecedented crisis in general practice, as well as the considerable demands on carers, it would be inappropriate and counter-productive to continue the interviews.

Lastly, the fact that the first author is also a carer, was important in informing the study design, although steps were taken to avoid any unintended researcher bias. For instance, the questionnaire was piloted with five carers from diverse caring roles. The interview questions were also reviewed by the research team and in collaboration with a carer and a GP. The framework analytical approach used in the analysis of the qualitative data, is also highly structured, with a clear audit trail, [31] while the lead researcher also kept a reflective journal throughout the interview process.

## Conclusion

This study has highlighted some of the possible reasons why the objectives of the National Carers Strategy [21], with respect to the identification and support of carers in community healthcare settings such as general practice, have remained largely unmet. Carers in this study were experiencing high levels of psychological distress, as well as important psychological barriers to help seeking, and, overall, they reported a low level of satisfaction with the supports they were receiving. GPs were unaware of the National Carers Strategy, were unclear about their role vis-à-vis carers and lacked training and resources to be able to identify, assess or support them in general practice/primary care. All of the indications are that carer numbers will continue to rise, [1] so it is critical that a robust process is put in place for the identification, signposting and support of family carers in general practice and primary care settings. Conversely, and as outlined earlier, general practice workloads are also set to increase [18], so policy makers and practice initiatives need to take into account the views of all stakeholders, including carers, and the challenges that they face, particularly in the context of such growing demands.

The changing needs along the care trajectory, suggest that carer identification, assessment and supports should be offered from the point of diagnosis of the care recipient, and a regular review process implemented thereafter. Further research could explore the extent to which the overburdening of family carers with ‘over medicalised’ tasks is present, and how parameters may be identified to help manage this issue. Interventions/initiatives that promote carer identification from the perspective of the carer, also need to be futher investigated, as well as ways to provide more resources and referral routes for GPs, including, for example, social prescribing. Social prescribing for family carers was recently noted by NICE to be an important knowledge gap [34] with no data currently available on its implementation or effectiveness.

The next stage of the larger multi-stage CHERISH project, currently underway and informed by the findings reported here, involves: (a) the development, piloting and evaluation of a brief educational workshop for GPs; (b) the development of an educational webinar (and attendant ‘Practice Points’ guidelines) for GPs to provide guidance to assist them in identifying, assessing and supporting carers; and (c) the design and delivery of an educational resource for family carers aimed at helping them to better address their issues related to carer identity and self-care, as well as their communication with GPs and other HCPs. The GP and carer-focused initiatives/resources have been co-designed, and will be co-delivered by both GPs and carers in line with public and patient involvement (PPI) guidelines [54]. Further information will be made available on these at a later date.

## References

[1] Spotlight (2019) Mind the care gap. *Exposing the health system’s vulnerability to the gap between family care provision and anticipated demand*. https://data.oireachtas.ie/ie/oireachtas/libraryResearch/2019/2019-04-02_spotlight-mind-the-care-gap-exposing-the-health-system-s-vulnerability-to-the-gap-between-family-care-provision-and-anticipated-demand_en.pdf. Accessed 7 Jan 2022

[2] Eurocarers (2021) European Association working for carers. https://eurocarers.org/about-carers/. Accessed 9 Feb 2022

[3] CSO (2016) Census of Population 2016 – Profile 9 Health, Disability and Carers. https://www.cso.ie/en/releasesandpublications/ep/p-cp9hdc/p8hdc/p9cr/. Accessed 7 Feb 2022

[4] Family Carers Ireland (2020) The state of caring. https://familycarers.ie/media/2022/family-carers-ireland-state-of-caring-2020.pdf. Accessed 7 Jan 2022

[5] Lefranc A, Perol D, Plantier M (2017). Assessment of informal caregiver’s needs by self-administered instruments: a literature review. Eur J Public Health.

[6] Thomas GP, Saunders CL, Roland MO, Paddison CA (2015). Informal carers’ health-related quality of life and patient experience in primary care: evidence from 195,364 carers in England responding to a national survey. BMC Fam Pract.

[7] Embracing Carers (2017) Carer report: embracing the critical role of caregivers around the world. https://www.embracingcarers.com/en/home.html. Accessed 5 Feb 2022

[8] OECD (2017) Informal carers, in health at a glance. https://www.oecd-ilibrary.org/sites/a80d9f62-en/index.html?itemId=/content/component/a80d9f62-en. Accessed 7 Feb 2022

[9] Chantal S, Satinder K, Tony K (2002) Psychosocial issues. Who cares for the carers? The district nurse perspective. Fam Pract 19(1):29 10.1093/fampra/19.1.29 11818347

[10] Parmar J, Anderson S, Abbasi M (2020). Support for family caregivers: a scoping review of family physician’s perspectives on their role in supporting family caregivers. Health Soc Care Community.

[11] Royal College of General Practitioners (2014) Involving and supporting carers and families : an educational framework and learning resource for GPs and primary care teams. RCGP, London, UK. Available from: https://www.oxfordhealth.nhs.uk/library/wp-content/uploads/sites/3/Involving-and-Supporting-Carers-and-Families-RCGP-January-20141.pdf. Accessed 7 Jan 2022

[12] Peters M, Rand S, Fitzpatrick R (2020). Enhancing primary care support for informal carers: a scoping study with professional stakeholders. Health Soc Care Community.

[13] Häikiö K, Cloutier D, Rugkåsa J (2020). Is health literacy of family carers associated with carer burden, quality of life, and time spent on informal care for older persons living with dementia?. PLoS ONE.

[14] Burridge L, Mitchell G, Jiwa M, Girgis A (2017). Helping lay carers of people with advanced cancer and their GPs to talk: an exploration of Australian users’ views of a simple carer health checklist. Health Soc Care Community.

[15] Smith PD, Martin B, Chewning B (2018). Improving health care communication for caregivers: a pilot study. Gerontol Geriatr Educ.

[16] Riffin C, Wolff JL, Estill M (2020). Caregiver needs assessment in primary care: views of clinicians, staff, patients, and caregivers. J Am Geriatr Soc.

[17] Fisher R, Parmar J, Duggleby W (2020). Health-care workforce training to effectively support family caregivers of seniors in care. Can Geriatr J.

[18] Crosbie B, O’Callaghan ME, O’Flanagan S (2020). A real-time measurement of general practice workload in the Republic of Ireland: a prospective study. Br J Gen Pract.

[19] Doctors of BC (2016) Organizing your practice to support family caregivers a toolkit for doctors. https://www.doctorsofbc.ca/sites/default/files/family_caregiver_resource_guide_for_physicians_-_toolkit.pdf. Accessed 5 Feb 2022

[20] Northern Sydney Local Health District (2019) Think patient – think carer supporting and involving carers, a guide for general practitioners and primary care teams. https://www.nslhd.health.nsw.gov.au/carer/documents/NS11895-E%20GP%20Guide%20-%20Think%20Patient%20-%20Think%20Carer.pdf. Accessed 4 Feb 2022

[21] Department of Health (2012) National carers’ strategy. recognised, supported, empowered. https://assets.gov.ie/10945/d62cf66f0a8f442bb594bbe0b48ef6ad.pdf. Accessed 8 Jan 2022

[22] Creswell JW (2007). Designing and conducting mixed methods research.

[23] Kutner G (2001) AARP caregiver identification study. AARP Research, February 2001

[24] Burrows A, Gannon K (2013). An evaluation of health and well-being checks for unpaid carers. Journal of integrated care (Brighton, England).

[25] Joseph S, Becker S, Elwick H, Silburn R (2012). Adult carers quality of life questionnaire (AC-QoL): development of an evidence-based tool. Ment Health Rev J.

[26] McKinn S, Bonner C, Jansen J, McCaffery K (2015). Recruiting general practitioners as participants for qualitative and experimental primary care studies in Australia. Aust J Prim Health.

[27] Family Carers Ireland (2020) Caring through COVID - life in lockdown. Family Carers Ireland. Dublin Ireland. https://familycarers.ie/media/1394/caring-through-covid-life-in-lockdown.pdf. Accessed 13 Apr 2022

[28] Homeniuk R, Collins C (2021). How COVID-19 has affected general practice consultations and income: general practitioner cross-sectional population survey evidence from Ireland. BMJ Open.

[29] Poudel P, Griffiths R, Wong VW (2020). Perceptions and practices of general practitioners on providing oral health care to people with diabetes - a qualitative study. BMC Fam Pract.

[30] Wichmann AB, Dam HV, Thoonsen BA (2018). Advance care planning conversations with palliative patients: looking through the GP’s eyes. BMC Fam Pract.

[31] Gale NK, Heath G, Cameron E et al (2013) Using the framework method for the analysis of qualitative data in multi-disciplinary health research. BMC Med Res Methodol 13 10.1186/1471-2288-13-117 PMC3848812 24047204

[32] Ritchie J, Spencer L, Bryman A, Burgess RG (1994). Qualitative data analysis for applied policy research. Analyzing qualitative data.

[33] Collins C, Homeniuk R (2021). How many general practice consultations occur in Ireland annually? Cross-sectional data from a survey of general practices. BMC Fam Pract.

[34] NICE (2020) Supporting adult carers. https://www.nice.org.uk/guidance/ng150. Accessed January

[35] Onwumere J, Shiers D, Chew-Graham C (2016). Understanding the needs of carers of people with psychosis in primary care. Br J Gen Pract.

[36] Jones R, Mackenzie A, Greenwood N (2012). General practitioners, primary care and support for carers in England: can training make a difference?. Health Soc Care Community.

[37] Carers Trust Wales (2019) Good practice approaches to supporting carers in Wales. Carers Trust Wales, Wales, UK. Available from: https://carers.org/downloads/resources-pdfs/good-practice-approaches-wales/good-practice-approaches-to-supporting-carers-in-wales.pdf. Accessed 5 Feb 2022

[38] Røen I, Stifoss-Hanssen H, Grande G et al (2019) Supporting carers: health care professionals in need of system improvements and education - a qualitative study. BMC Palliat Care 18(1):N.PAG-N.PAG. 10.1186/s12904-019-0444-3 10.1186/s12904-019-0444-3 PMC6636145 31311536

[39] Straub RO (2019) Health psychology: a biopsychosocial approach, 6th ed, edn. Macmillan International Higher Education, New York, NY

[40] Carduff E, Finucane A, Kendall M (2014). Understanding the barriers to identifying carers of people with advanced illness in primary care: triangulating three data sources. BMC Fam Pract.

[41] Family Carers Ireland (2022) Counting carers: carer prevalence in Ireland Working paper 1. https://familycarers.ie/media/2381/counting-carers-carer-prevalence-in-ireland.pdf. Accessed 13 Apr 2022

[42] Cronin M, McGilloway S (2020) Promoting a ‘Think Carer’ approach in health and social care services: Identifying as a carer – Why is it important? Frontline Irish Voice of Intellectual Disability (online), Issue 116. Available from: http://frontline-ireland.com/promoting-a-think-carer-approach-in-health-and-social-care-services-identifying-as-a-carer-why-is-it-important/. Accessed 6 Feb 2022

[43] Montgomery RJV (2007) Family caregiver. In: Blackburn JA, Dulmus CN (ed). Handbook of gerontology: evidence-based approaches to theory, practice, and policy, chapt IV, 588. Hoboken, NJ, US: John Wiley & Sons, Inc, pp 426–454

[44] Eifert EK, Adams R, Dudley W, Perko M (2015). Family caregiver identity: a literature review. Am J Health Educ.

[45] Carduff E, Jarvis A, Highet G (2016). Piloting a new approach in primary care to identify, assess and support carers of people with terminal illnesses: a feasibility study. BMC Fam Pract.

[46] Royal College of General Practitioners, The Princess Royal Trust (2013) Supporting carers: an action guide for general practitioners and their teams (2nd ed). 19, 26:34–19, 26, 34

[47] Parmar J, Anderson S, Abbasi M (2020). Support for family caregivers: a scoping review of family physician’s perspectives on their role in supporting family caregivers. Health Soc Care Community.

[48] Reinhard S (2019) Home alone revisited: family caregivers providing complex care. Innov Aging 3(Supplement_1):S747-S748. 10.1093/geroni/igz038.2740

[49] Dow B, McDonald J (2007). The invisible contract: shifting care from the hospital to the home. Aust Health Rev.

[50] White S, Hart N, Lewis S (2021). Engaging carers in co-design: development of the carer readiness tool. Int J Integr Care.

[51] Lafferty A, O’Sullivan D, O’Mahoney P (2016). Family carers’ experiences of caring for a person with intellectual disability.

[52] Haslam SA, McGarty C (2014). Research methods and statistics in psychology.

[53] Archer R, Elder W, Hustedde C (2008). The theory of planned behaviour in medical education: a model for integrating professionalism training. Med Educ.

[54] Ignite Patient and Public involvement in research: values and principles framework (2022) HSE Research and Development, Dublin, Ireland. https://hseresearch.ie/patient-and-public-involvement-in-research-ignite-network/. Accessed 20 Apr 2022

